# Atomically flat and uniform relaxed III–V epitaxial films on silicon substrate for heterogeneous and hybrid integration

**DOI:** 10.1038/s41598-017-15025-0

**Published:** 2017-11-07

**Authors:** Martin Holland, Mark van Dal, Blandine Duriez, Richard Oxland, Georgios Vellianitis, Gerben Doornbos, Aryan Afzalian, Ta-Kun Chen, Chih-Hua Hsieh, Peter Ramvall, Tim Vasen, Yee-Chia Yeo, Matthias Passlack

**Affiliations:** 1 TSMC R&D Europe B.V., Kapeldreef 75, 3001 Leuven, Belgium; 2 TSMC, No. 8. Li-Hsin Rd. VI, Hsinchu Science Park, Hsinchu, 300-78 Taiwan, R.O.C.

## Abstract

The integration of III-V semiconductors on silicon (Si) substrate has been an active field of research for more than 30 years. Various approaches have been investigated, including growth of buffer layers to accommodate the lattice mismatch between the Si substrate and the III-V layer, Si- or Ge-on-insulator, epitaxial transfer methods, epitaxial lateral overgrowth, aspect-ratio-trapping techniques, and interfacial misfit array formation. However, manufacturing standards have not been met and significant levels of remaining defectivity, high cost, and complex integration schemes have hampered large scale commercial impact. Here we report on low cost, relaxed, atomically smooth, and surface undulation free lattice mismatched III-V epitaxial films grown in wide-fields of micrometer size on 300 mm Si(100) and (111) substrates. The crystallographic quality of the epitaxial film beyond a few atomic layers from the Si substrate is accomplished by formation of an interfacial misfit array. This development may enable future platforms of integrated low-power logic, power amplifiers, voltage controllers, and optoelectronics components.

## Introduction

Many approaches have been pursued to integrate III-V semiconductors on Si substrate^1–20^. Confinement of defects at heteroepitaxial interfaces using the concept of interfacial misfit array formation (IMF) was proposed earlier^20,21^. On Si substrate, epitaxial growth of III-V materials (e.g. AlSb) by molecular beam epitaxy was demonstrated using the IMF concept, however, significant levels of surface undulations and roughness remained even after growth of thick layers exceeding hundreds of nanometers or more. Here, we employ the IMF concept to demonstrate growth of lattice mismatched epitaxial III-V films (InAs) which are relaxed, atomically smooth, and surface undulation free in flat bottom shallow trench isolation (STI) defined wide-fields of micrometer size on Si(100) and (111) 300 mm substrates. The InAs layers with a thickness between 7 and 40 nm are fabricated by selective area low-cost metal organic chemical vapor deposition (MOCVD) using a three step growth technique involving a low temperature nucleation step to form a seed layer, a coalescence annealing step at higher temperature to form a high quality ultrathin template layer, and a final high temperature fill step to form a layer of target thickness.

There has been significant process in the epitaxy of GaN on various substrates recently and the mechanism bears some resemblance to the work reported here (seed layer consisting of GaN islands flowed by island coalescence) but there are inherent differences with the present work. GaN on Si promotes the reduction of threading dislocation density (TDD) from 10^10^ cm^−2^ to 10^8^ cm^−2^ over large areas for LED applications^22^. This reduction of TDD is important in the LED field. The focus of this work is CMOS which requires much lower TDD (<10^3^ cm^−2^) for devices typically on the scale of 10–100 nm.

This work builds on IMF of antimonides on Si and GaAs and the GaN on Si work cited above but is novel in many ways. The aim here is focused on CMOS so requires very low defectivity but can use restricted areas (2 μm^2^) where many CMOS logic devices can be built. The application space is heterogeneous CMOS devices on different areas of the same 300 mm wafer. The epitaxy technique used is low temperature MOCVD compared to MBE for antimonides on Si and high temperature MOCVD for GaN on Si. These differences relate directly to the density of vertically propagating defects. The achievement of an InAs channel CMOS device with a high I_on_/I_off_ ratio grown on Si emphasizes the differences with previous work as does the ability to grow atomically flat InAs on Si (111) over large areas (previous attempts to grow planar InAs on Si using multiple anneals gave a rougher surface^23^). The flat InAs on Si(111) has direct technology applications as it avoids CMP (chemical-mechanical polishing) in a device integration process reducing cost and complexity.

**Figure 1:**
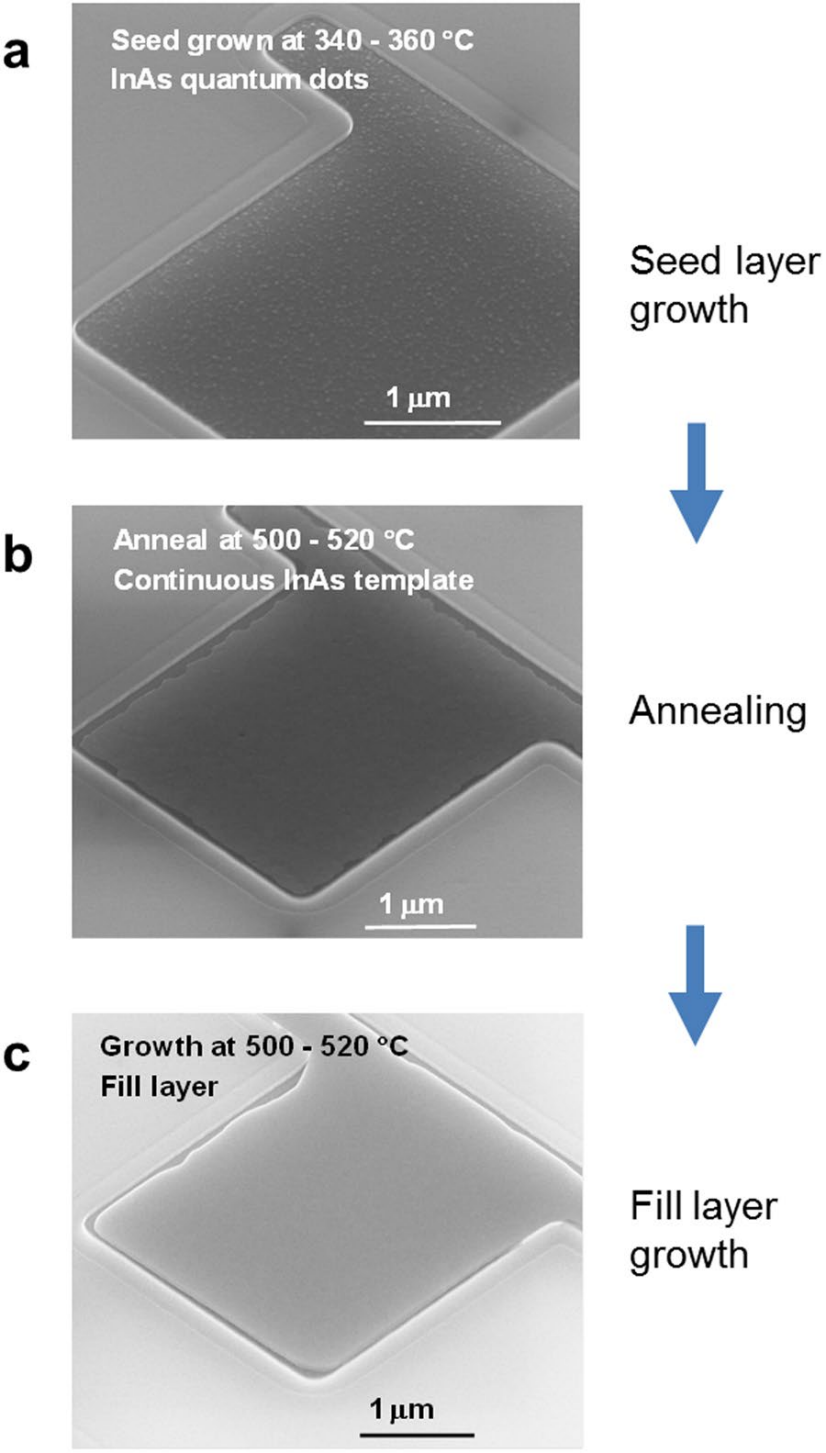
SEM images of InAs epitaxial layers directly grown on Si(111) showing stages in the epitaxial growth process. (**a**) The InAs seed grown at 340–360 °C shows many InAs quantum dots about 20–30 nm in diameter. (**b**) Annealing at 500–520 °C causes the dots to merge into a single two-dimensional InAs template layer. (**c**) Fill layer growth provides a layer of target thickness.

We used scanning electron microscopy (SEM), cross sectional high resolution scanning transmission electron microscopy (HR-STEM), and atomic force microscopy (AFM) to characterize the InAs layers grown in wide-fields of micrometer size on Si substrate. Figure 1a shows a top view SEM image of InAs on Si(111) before annealing. Many InAs quantum dots about 20–30 nm in diameter are visible. Annealing causes the quantum dots to merge into a single two-dimensional InAs epitaxial template (Fig. 1b). Finally, an InAs fill step provides an InAs layer of desired thickness (Fig. 1c).

**Figure 2:**
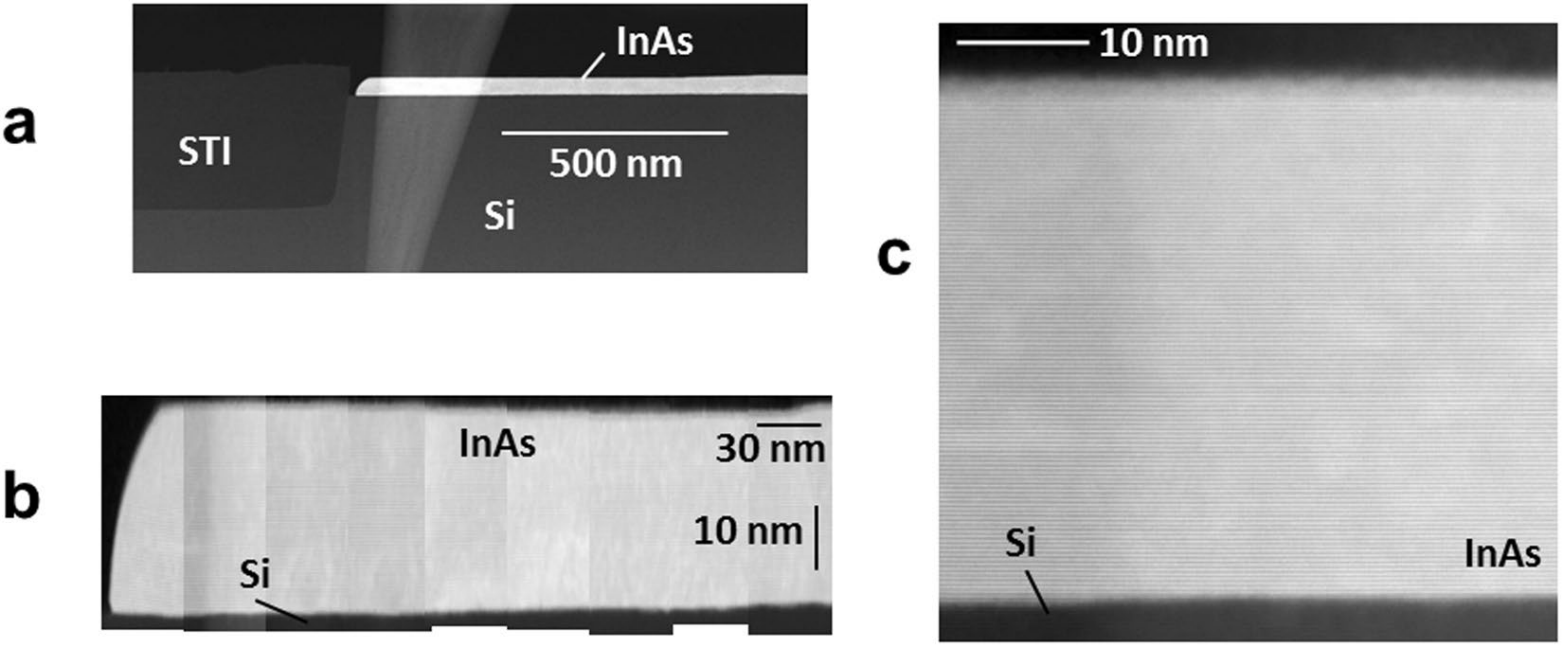
Cross sectional STEM analysis of an as grown 33 nm thick epitaxial InAs layer in a wide-field of micrometer size on a Si(111) substrate. (**a**) Low resolution TEM image of an approximately 1 μm long section providing an overview of the investigated epitaxial structure including STI and Si substrate showing an undulation free InAs surface. (**b**) Individual HR-STEM images of 9 adjacent segments are acquired with high resolution, the complete image is assembled, and the image is scaled along the lateral axis by a factor of 3 and maintaining the vertical scale to obtain a publishable size image while simultaneously preserving image information. The length of the assembled section is about 340 nm demonstrating the crystallographic quality of an extended region. The rounded shape at the edge of the epitaxial layer is a typical observation. (**c**) Original size HR-STEM image of one of the segments in (**b**).

Figure 2 shows a cross sectional STEM analysis of an as grown 33 nm thick epitaxial InAs layer in a wide-field of micrometer size on a Si(111) substrate. A low resolution TEM image provides an overview of the investigated epitaxial structure including STI and Si substrate showing an undulation free, smooth, and uniform InAs surface for a section of about 1 μm length (Fig. 2a). In order to elucidate the crystallographic quality of an extended region, individual images of 9 adjacent segments are acquired with high resolution, the complete image is assembled, and the image is scaled along the lateral axis by a factor of 3 and maintaining the vertical scale to obtain a publishable size image while simultaneously preserving image information (Fig. 2b). Since some detailed information contained in the high resolution images is difficult to observe in Fig. 2b, an original size HR-STEM image of one segment is added for completeness (Fig. 2c).

**Figure 3:**
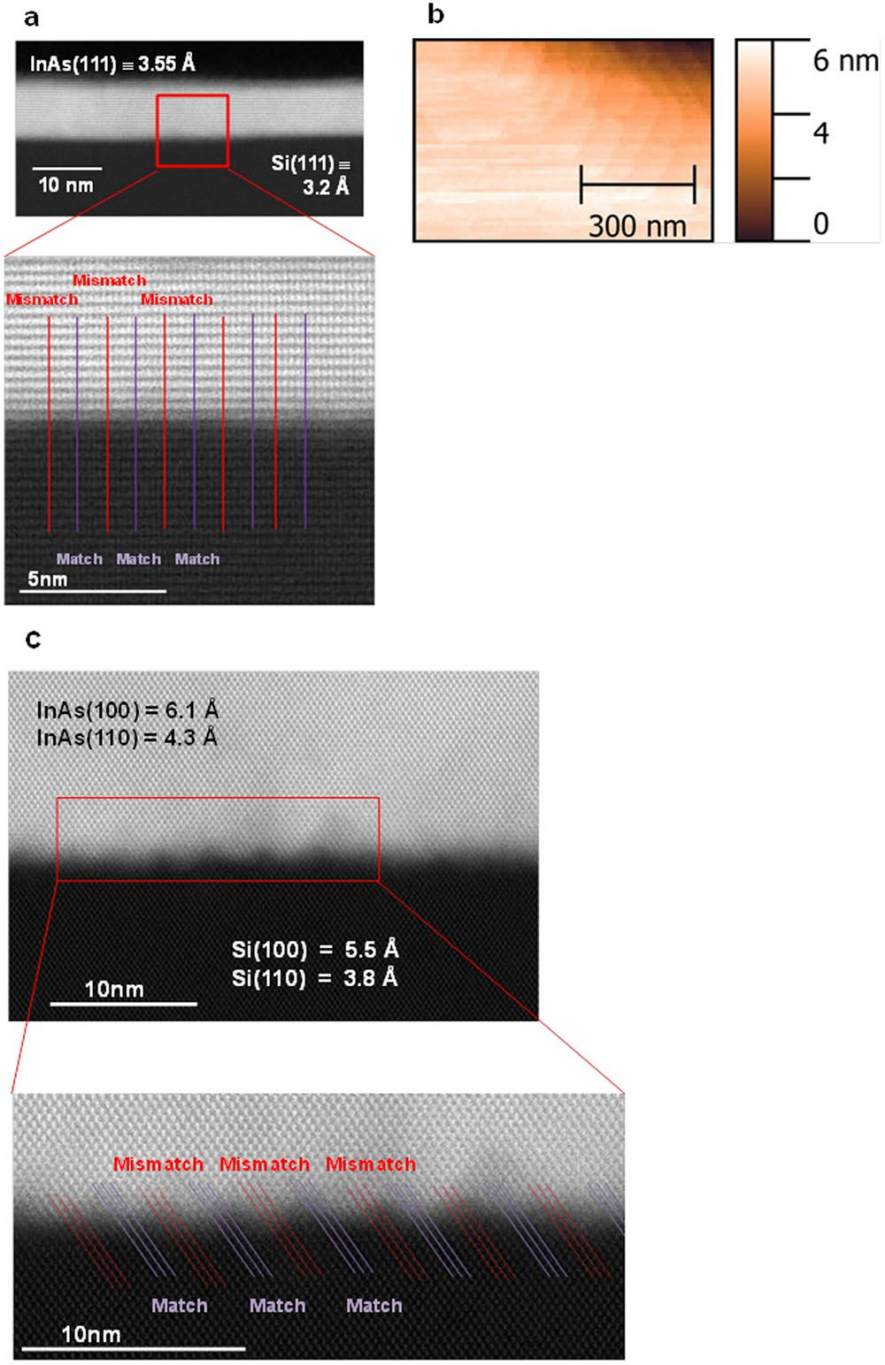
Cross sectional HR-STEM and AFM analysis of epitaxial InAs layers grown in a wide-field of micrometer size on Si substrate. (**a**,**b**) HR-STEM image illustrating the crystallographic quality beyond a few atomic layers from the Si substrate and lattice parameter of both Si substrate and InAs epitaxial layer and AFM surface scan with a root-mean-square roughness = 0.13 nm over a scan area of 1 μm × 0.5 μm of a 7 nm InAs layer grown in a 2 μm × 2.5 μm STI defined area on Si(111). The inset shows 9 InAs atoms for 10 Si atoms at the interface. The vertical (111) lattice constant correspond to the values of relaxed InAs and Si. (**c**) HR-STEM image of the interfacial region of a 40 nm InAs epitaxial layer grown in a 1 μm × 50 nm STI defined area on Si(100). The vertical (100) and horizontal (110) lattice parameters correspond to the values of relaxed InAs and Si. A periodic array of misfit dislocations at the Si-InAs interface accommodate the lattice mismatch (see inset) with 10 Si atoms accommodating 9 InAs atoms.

A further HR-STEM image illustrates the single-crystal and relaxed structure of the epitaxial film (Fig. 3a), and the AFM surface scan (Fig. 3b) depicts the atomically smooth surface morphology (root-mean-square roughness = 0.13 nm) of an as grown 7 nm InAs layer in a 2 μm × 2.5 μm wide-field STI defined area on Si(111). For epitaxy on Si(100), a HR-STEM image of the interface region of a 40 nm relaxed InAs epitaxial layer grown in a 1 μm × 50 nm STI defined area is shown in Fig. 3c illustrating the crystallographic quality of the InAs layer beyond a few atomic layers from the silicon substrate. For InAs on the Si(100) surface, the IMF dislocation array can be seen in Fig. 3c. Periodic undulations can clearly be seen at the interface. There is some evidence of periodic dark and light bands which indicate strain fields from bond bending around the dislocations. The misfit period is 5.4 nm which represents nine InAs lattice sites grown on ten Si atoms.

During the epitaxial process, the nucleation layer begins with quantum dots which will be crystalline and pseudomorphic. Further deposition causes the islands to coalesce and layer by layer growth to start. Undulations at the interface are a strain relief mechanism. The strain is accommodated more easily because of the additional surface area. The undulations lead to misfit dislocations that propagate parallel to the substrate. There is no evidence of vertical propagating defects (threading/screw dislocations). The presence of 90° misfit dislocations which move laterally, confined to the interface as the primary strain relief mechanism and the absence of 60° misfit dislocations associated with device damaging vertical defects is critical.

60° misfits have a number of causes: i) lower strain promotes 60° misfits. 90° misfits dominate at high strain^24^ so the highly mismatched Si-InAs interface will suppress 60° defects ii) Higher temperature provides the energy for 60° misfits to form^25^. In this work, the low growth temperatures used during the seed layer (350°C) are made possible by using TBA (tertiarybutylarsine) rather than AsH_3_. These low temperatures ensure In-As bond will remain strong, reducing the chance of vertically propagating defects. iii) Island boundaries on (100) surfaces can provide (111) planes that are the source of 60° misfits so the Si-InAs interface on (100) is more vulnerable to threading dislocations than the same interface on the (111) substrate.

In Fig. 3c, the low temperature epitaxy is sufficient to suppress vertical dislocations in the device area. The lattice constant above the interface in InAs is 6.1 Å [001] indicating the InAs is fully relaxed within a few atomic layers of the interface. The 11.5% mismatch is compensated by the IMF and associated undulations.

In contrast to the (100) Si-InAs interface, the same interface on a (111) surface shows no signs of undulations or strain-related dark and light bands. This suggests the bonding reconfiguration required to form the IMF and compensate for the lattice mismatch on (111) is less. The monolayer separation above the interface in InAs is 3.55 Å [111] compared to 3.2 Å in the Si[111] indicating the InAs is fully relaxed a few atomic layers from the interface.

**Figure 4:**
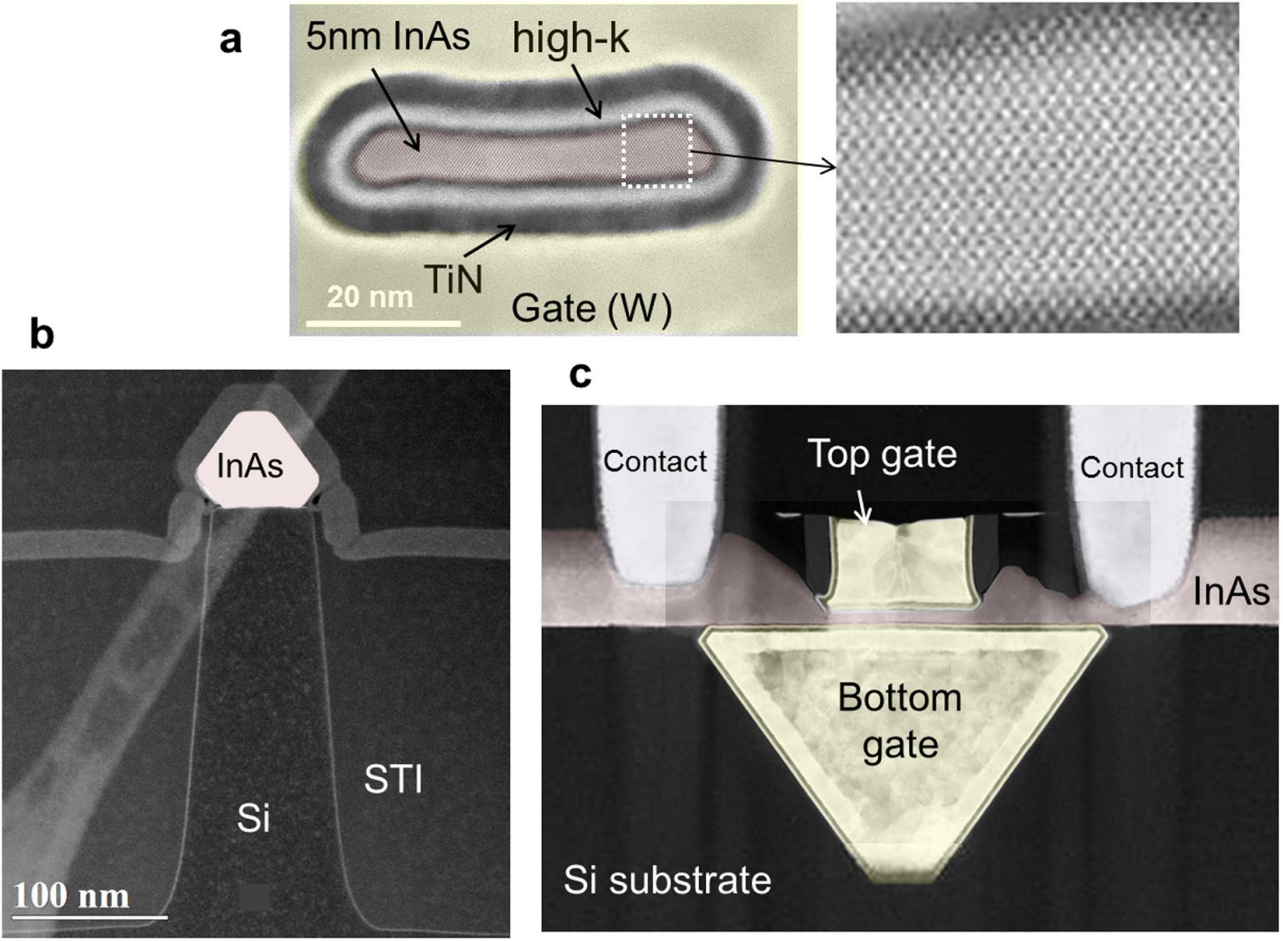
STEM cross sectional images of fabricated NW FETs with InAs channel grown in a wide-field of micrometer size directly on Si(100). (**a**,**b**) STEM image perpendicular to the NW in the gate and source/drain areas, respectively. (**c**) TEM image along the NW showing gate, source, and drain contacts.

**Figure 5:**
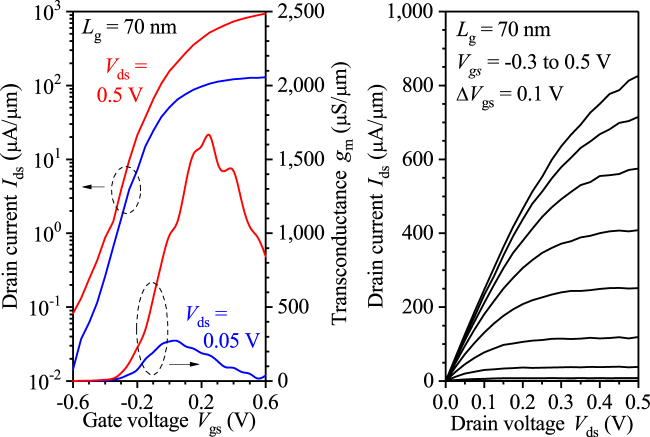
Transfer and output characteristics of NW FET with a 4–5 nm high InAs channel directly grown on Si(100). (**a**) Peak transconductance *g*_m_ = 1,700 μS/μm, subthreshold swing *SS* = 114 mV/dec, and *Q* = *g*_m_/*SS* of 15.4 are measured for *L*_g_ of 70 nm at *V*_ds_ = 0.5 V.

To verify the use of the epitaxy process described above for device applications, a InAs nanowire field-effect transistor was fabricated in InAs wide fields by adopting a device flow which was developed earlier for NW FETs^26^ using conventional aspect-ratio-trapping techniques^18^. HR-STEM cross sectional images of fabricated NW FETs with InAs channel directly grown on Si(100) are shown perpendicular to the InAs NW in the gate area (Fig. 4a) and source/drain contact area (Fig. 4b) as well as along the NW (Fig. 4c). For this device, the NW height *H*_NW_ = 4–5 nm and width *W*_NW_ = 40 nm resulting in a total gate periphery of 90 nm. Transfer (*I*_ds_-*V*_gs_) and output characteristics (*I*_ds_-*V*_ds_) of NW FETs with InAs channel directly grown on Si(100) are shown for gate lengths *L*_g_ of 70 nm (Fig. 5). The measured peak transconductance = 1700 μS/μm and subthreshold swing = 114 mV/dec for *L*_g_ = 70 nm at *V*_ds_ = 0.5 V.

We have devised a method based on the IMF concept^20,21^ which allows growth of lattice mismatched, relaxed epitaxial InAs films of high crystallographic quality beyond a few atomic layers from the Si substrate. These films are atomically smooth in STI defined wide-fields on Si(100) and Si(111) 300 mm substrates. These findings and envisioned extensions of the method to other III-Vs and lattice mismatched system may enable future platforms for heterogeneous and hybrid integration of low-power logic, power amplifiers, voltage controllers, and optoelectronics components. This platform is lower cost (growth of thick buffer layers is not required), provides more reliable and higher performance devices (low thermal conductivity buffer layers are absent), and enables easier and more flexible fabrication (large step heights are avoided). This application space is in contrast to that of nanowires where III-V growth has been demonstrated previously.

## Methods Summary

Atomically flat, epitaxial InAs films of high crystallographic quality beyond a few atomic layers from the on-axis Si substrate (no miscut) in flat bottom shallow trench isolation (STI) defined wide-fields of micrometer size on Si(100) and (111) 300 mm substrates, have been demonstrated using selective area MOCVD. The nucleation of the first few monolayers of III-V on Si and the condition of the Si surface are critical to the defectivity of the III-V material. Antiphase domain defects (APDs) can be avoided by converting the single steps to double atomic steps by baking at temperatures >900 °C and annealing in hydrochloric vapor at lower temperatures. This method also leads to *a flat bottom surface* at the trench bottom. Subsequently, plasma etch/annealing cycles are used followed by a substrate anneal at 520–540 °C to terminate the Si surface with As atoms. The substrate temperature is then lowered to 340–360 °C to deposit the InAs quantum dot seed layer. Subsequently, the substrate temperature is raised to 500–520 °C to transform the InAs quantum dots into a continuous InAs template layer. A high TBA flow is required during the anneal to prevent faceting. Finally, the InAs fill layer is grown at a temperature of 500–520 °C to target thickness. For device processing, optional chemical-mechanical planarization (CMP) can be introduced to level-off the InAs top surface with the STI surface. After the InAs channel formation, the integration process follows a standard replacement-gate (RPG) FinFET integration flow^26^, up to the replacement gate stage. The Si under the InAs channel is removed by a selective wet chemistry, in this case TMAH. Afterwards, integration follows standard back-end-of-line processing.

### Data availability statement

Materials or information can be provided to readers upon request. TSMC is a for-profit company.
